# Comparison of different protocols of Morris water maze in cognitive impairment with heart failure

**DOI:** 10.1002/brb3.1519

**Published:** 2020-01-16

**Authors:** Ziwen Lu, Tao Yang, Lei Wang, Qi Qiu, Yizhou Zhao, Aiming Wu, Tong Li, Wenkun Cheng, Baofu Wang, Yang Li, Jingjing Yang, Mingjing Zhao

**Affiliations:** ^1^ Key Laboratory of Chinese Internal Medicine of Ministry of Education and Dongzhimen Hospital Beijing University of Chinese Medicine Beijing China; ^2^ Department of Pharmacy Beijing Anzhen Hospital Capital Medical University Beijing China

**Keywords:** cognitive impairment, heart failure, Morris water maze, working memory

## Abstract

**Aim:**

This study aimed to find a more sensitive and systematic behavioral evaluation protocol to evaluate the cognitive impairment in rats with heart failure (HF).

**Methods and results:**

An HF rat model was built by ligating the left anterior descending coronary artery. The cardiac function and structure were detected using echocardiography. Myocardial histopathological changes were observed by nitro blue tetrazolium and hematoxylin–eosin staining. The cognitive functions were evaluated using the acquisition task, probe trial, reversal test, and matching‐to‐sample test of the Morris water maze. In the probe trial, the number of times the rats in the model group crossed the platform site significantly decreased compared with that in the sham group. In the reversal test, the average latency was significantly longer in the sham group compared with the model group in the first trial but was shorter in the second and third trials. In the matching‐to‐sample test, the average latency of Trial1 increased significantly in the model group compared with the sham group, while no obvious difference was observed in Trial2. Therefore, the difference in the average latency between Trial1 and Trial2 of the model group was significantly larger.

**Conclusions:**

The cognitive impairment in rats with HF mainly reflected in the long‐term and working memory, spatial learning, and reversal learning ability. The probe trial and reversal test in the water maze may be more sensitive and preferred to evaluate cognitive function after HF. These findings would provide a brief evaluation protocol for further studies on the relationship between cognitive function and HF.

## Graphical abstract

This study aimed to find a more sensitive and systematic behavioral evaluation protocol to evaluate the cognitive impairment in rats with heart failure (HF).The cognitive impairment in rats with HF mainly reflected in the long‐term and working memory, spatial learning and reversal learning ability.

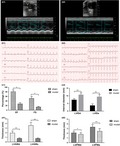

## 1 INTRODUCTION

Clinically, people with chronic heart failure (CHF) frequently have mental health problems, such as cognitive impairment and depression (Almeida et al., 2012). Cognitive disorder prevalent in people with heart failure (HF) ranges from 25% to 80% according to different studies (Davis & Allen, 2013; Pressler, 2008). The evidences have shown that HF is associated with mild cognitive impairment, which is marked by a decline in cognition beyond normal criteria expected for a person's age or educational level. Furthermore, cognitive dysfunction also contributed to higher mortality and hospitality in patients with CHF (Jennifer 2015, O'Donnell et al., 2012, Sauve, Lewis, Rickabaugh, & Pressler, 2009). Therefore, early detection and timely treatment are essential.

The cognitive impairment after CHF is divided into different domains of cognitive function, including memory, learning, executive function, processing speed, and fluid intelligence (Kindermann et al., 2012). Different from Alzheimer's disease and vascular dementia (VD), this kind of early cognitive impairment with HF is mild and subtle, and difficult to measure (Pressler, 2008). MCI is defined as a clinical stage between normal aging and mild dementia (Langa & Levine, 2014). Hence, no perfect assessment method existed to evaluate the cognitive function of patients with HF. Various methods were used to comprehensively evaluate the cognitive function in clinic, while the Montreal Cognitive Assessment (MoCA) and Mini‐Mental Status Examination (MMSE) were mostly used (Cameron, Worrall‐Carter, Page, Stewart, & Ski, 2013).

Similarly, in the basic research studies, assessment of the cognitive function of rats with HF is difficult, and only a few studies investigated the evaluation methods. Therefore, establishing a behavioral evaluation method is necessary. The Morris water maze is a universal method to evaluate the cognitive function of animals (Hooge & De Deyn, 2001). Several studies presented different and inconsistent results in evaluating the cognition of rats with HF using the typical Morris water maze method (Hong et al., 2013; Qin, Wang, Wang, Zhao, & LV, 2006). Therefore, the acquisition task was not sensitive enough to evaluate the cognitive impairment of rats with HF and could not detect the dysfunction of different cognitive domains.

In the present study, the left anterior descending coronary artery ligation surgery of the model rats was performed to build the HF model (Wu et al., 2017). After 60 days of the surgery, the cognition of rats with HF was tested through not only the spatial acquisition task and probe trial but also the more complex reversal task and matching‐to‐sample test of the Morris water maze. On the one hand, this study aimed to find a more sensitive and systematic behavioral evaluation protocol to evaluate the cognitive impairment of rats with HF. On the other hand, different tasks of the Morris water maze were jointly used to detect the damage to cognitive domains, including learning, memory, and executive function in rats with HF.

## 2 MATERIALS AND METHODS

### 2.1 Animals

Male Sprague–Dawley rats (body weight 240 ± 10 g) used in this study were sourced from Beijing Vital River Laboratory Animal Technology Co. Ltd [Animal license number: SCXK (Beijing) 2012–0001]. The animals were housed in humidity‐controlled (60% ± 10%) rooms at 24°C ± 1°C and maintained under a 12‐hr light–dark cycle. The animals were provided with standard diet and water. All animal experiments were approved by the Animal Care and Use Committee of Beijing University of Chinese Medicine.

### 2.2 Establishment of the HF model of rats

The HF model of rats was established, as discussed in a previous study (Wu et al., 2013). The rats were anesthetized with 1% solution of sodium pentobarbital (40 mg/kg) through intraperitoneal injection. Then tracheal intubation was performed via the oral cavity of rats, and the rats were connected to the ventilator. After opening the chest of the rat, the left anterior descending coronary artery was occluded between the pulmonary cone and the left atrial appendage under its origin 2–3 mm. However, in the sham operation group, the operation was conducted without ligation. A 12‐lead electrocardiogram (ECG) was performed both preoperatively and postoperatively. Successful ligation was confirmed by the pathological Q waves 6–8 (including I lead, AVL lead, and V1–V6 lead) shown in ECG 24 hr after the surgery. Penicillin was injected intraperitoneally every day in the following 3 days after the surgery to prevent infection. The echocardiographic examination was made to evaluate the cardiac function of rats, with an ejection fraction (EF) of less than 40% to be seen as a successful model.

### 2.3 Design and allocation

In this study, two experiments were conducted because two different kinds of Morris water maze protocols were used to evaluate the cognition of the HF model of rats. In experiment 1, the rats with successful coronary artery ligation after surgery were assigned to the model group while those without ligation were assigned to the sham operation group, with 15 rats in each group. After 60 days of the surgery, all rats underwent echocardiography examination. Then, the acquisition trial, probe trial, and reversal test of the Morris water maze were performed. Subsequently, the rats were sacrificed with hearts harvested for the following histopathological preparations. In experiment 2, all the experimental procedures were the same as experiment 1 except the matching‐to‐sample test of Morris water maze to evaluate cognition.

### 2.4 Electrocardiography and echocardiography

The electrocardiography was performed 30 min and 24 hr after the surgery while the echocardiography was performed 60 days after the surgery, which was used to evaluate the heart function. The rats were injected with 1% solution of sodium pentobarbital (40 mg/kg) and fixed on their backs on boards with skin cleaned. After electrode needle insertion under the skin of the upper limb, lower limb, and chest of rats, the 12‐lead ECG was performed.

The examination of echocardiography was carried out using the Vevo 2100 Imaging System Ultrasonic Diagnostic Equipment (VisualSonics). The ultrasonic probe with a 15‐MHz high‐frequency linear array transducer was placed on the left side of the sternum, showing the parasternal long‐axis view. The heart function was measured using the M‐curve, guided by the two‐dimensional ultrasonic image. The main parameters consisted of EF (%) and fractional shortening (FS, %).

### 2.5 Morris water maze tests

#### 2.5.1 Acquisition trial, probe trial, and reversal test of the Morris water maze

The Morris water maze apparatus was purchased from Shanghai Mobile Datum Information Technology and consisted of a 1.6‐m diameter pool, which was divided into four quadrants (NE, SW, SE, and NW). The pool was surrounded by light blue curtains with attached different shapes (such as triangle, square, circle, and pentagon). The tank was suffused with the 27‐cm‐deep water level, which was mixed with 30 ml of ink to obscure visual cues. The temperature of the water was maintained at 23°C–24°C. A platform 12 cm in diameter was hidden 1–2 cm below the surface of the water. The experimental room was soundproof and without direct light.

The rats were firstly pretrained for 1 day. Experiment 1 comprised three phases: acquisition trial, probe trial, and reversal test. In the acquisition trial, the rats were placed on the platform located in the SW quadrant for 5 s and then randomly released into the water from the other three quadrants (NW, SW, NE) with the head toward the wall of the tank. The time was limited to 120 s per trial, and the activities were recorded. If the rats did not find the platform in 120 s, they were guided to the platform and placed on it for 5 s (this adaptation training was performed only once). Three trials were performed per day for three consecutive days with each rat so that each rat underwent nine trials in all for this phase. In the probe trial, 24 hr after the acquisition trial, the platform was removed and the releasing point was similarly to the acquisition trial, while number of times the rats crossed the platform site in 120 s was recorded. In the reversal test, the platform was moved to the opposite quadrant (NE) and the rats were released into the water as done on the first day of the acquisition trial. The swimming time and path were recorded.

#### 2.5.2 Experiment 2: Matching‐to‐sample test of the Morris water maze

In experiment 2, the water maze apparatus and experimental situation were the same as in experiment 1. The rats were pretrained for 1 day before the official test. Each rat received four pairs of trials every day, for five consecutive days. The locations of platform and entry points were different and randomly changed between each pair of trials. Each pair of trials consisted of Trial1and Trial2. In each pair of Trial1, the rats were placed in the platform for 5 s first and then into the water with heads toward the wall of the tank; the time was limited 120 s to find the platform. If the rats did not find the platform within this time, they were guided to the platform and placed on it for 30 s (this adaptation training was performed only for once). After a 15‐s intertrial interval, the rats were placed in the water again in Trial2, and the location of the platform and entry points were the same as in Trial1. The swimming time and path were recorded.

### 2.6 Nitro blue tetrazolium staining

After echocardiography and behavioral examination, the hearts of the rats were cut uniformly into five 1‐mm‐thick transverse sections. Then, they were put in 0.1% nitro blue tetrazolium (NBT) stains, colored for 6–8 min in an incubator maintained at 37°C, and kept away from light. The normal myocardial tissues showed a uniform bluish‐purple stain while those in the infarction area presented a gray‐white stain, which formed a sharp contrast with the normal blood supplying area and made it easy to locate and measure. The stained myocardial tissues were photographed immediately.

### 2.7 Hematoxylin and eosin staining

The heart samples of the rats were fixed in 4% paraformaldehyde solution. Then, 3‐mm‐thick heart tissue was cut from the maximum transverse diameter of the heart. The tissues were dehydrated, cleared, and embedded in paraffin. Then, 4‐μm‐thick slices were cut and stained with hematoxylin and eosin (HE). The changes in myocardial morphology were observed under a light microscope (×400).

### 2.8 Statistical analysis

All statistical analyses were performed using SPSS version 20. All continuous variables were expressed as mean ± standard deviation. If the distribution of the variable was normal, the *t* test was used, otherwise the Mann–Whitney *U* test was used. For behavioral data, the significance of differences was assessed by ANOVA for repeated measures. A value of *p* < .05 was considered statistically significant.

## 3 RESULTS

### 3.1 Decline in the cardiac function

**Figure 1:**
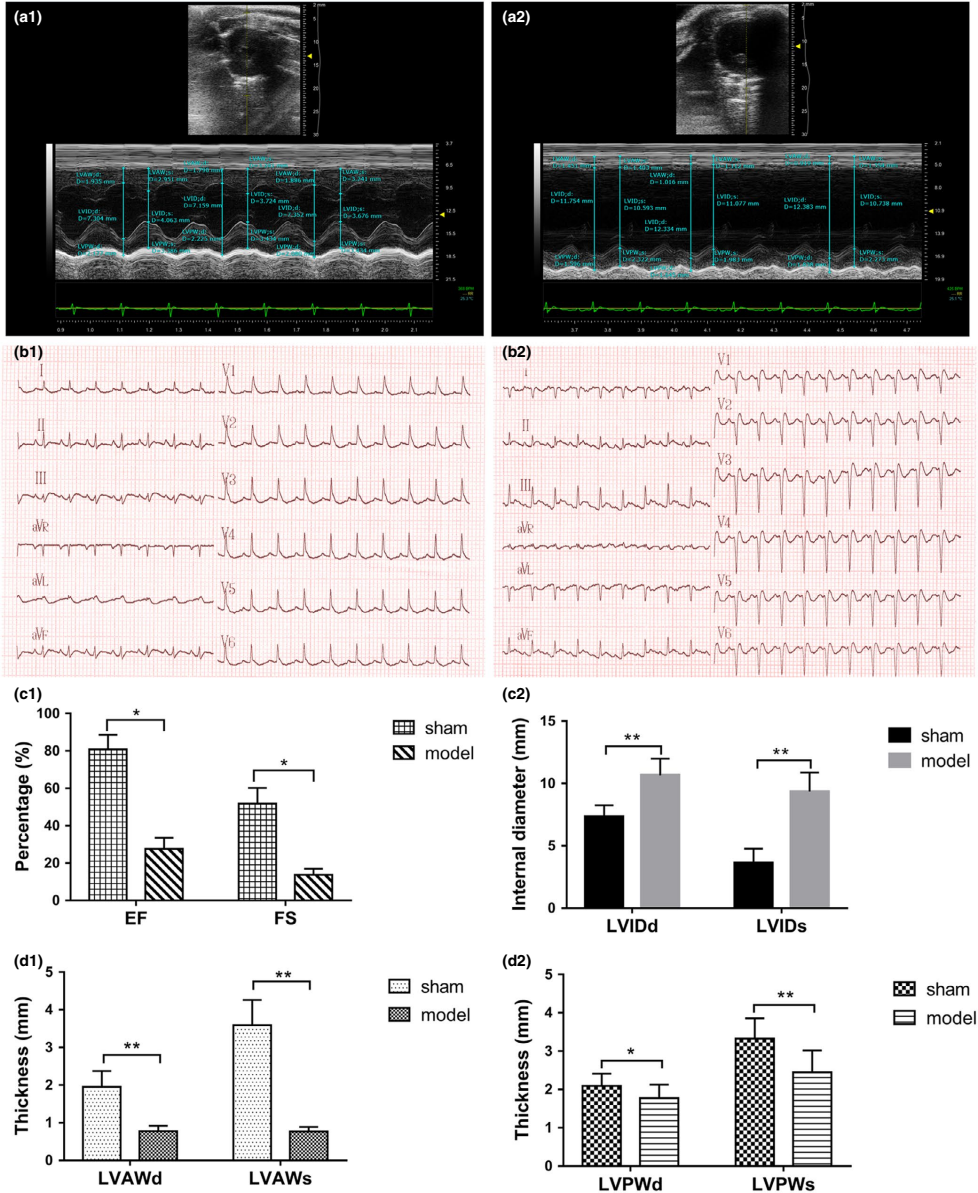
Echocardiography images of the sham group (a1) and the model group (a2) for 60 days. ECG recordings of the sham group (b1) and the model group (b2) for 60 days. EF and FS of the sham and model groups for 60 days (c1). Left ventricular internal diameter of the sham and model groups for 60 days (c2). The ventricular wall thickness of the sham and model groups for 60 days (d1 and d2). *p < .05, **p < .01

The results of electrocardiography and ECG revealed the cardiac structure and function. In the model group, the results of ECG showed significant pathological Q waves 24 hr after ligation (Figure 1b2), which indicated the transmural MI (myocardial infarction) of rats. As shown in Figure 1a2, the ventricular contraction movement of normal rats was in the shape of waves, which were straightened and disappeared into the anterior wall in the model group. This indicated the weakness of the contraction movement. Furthermore, Figure1c1 shows that the EF and FS decreased significantly in the model group compared with the sham group (*p* < .05). The LVIDd and LVIDs in the model group were significantly increased compared with the sham group (C2). And the left ventricular wall thickness of model group was obviously thinner than the sham group (D1 and D2).

### 3.2 Myocardial histopathological findings

#### 3.2.1 Results of NBT staining

**Figure 2:**
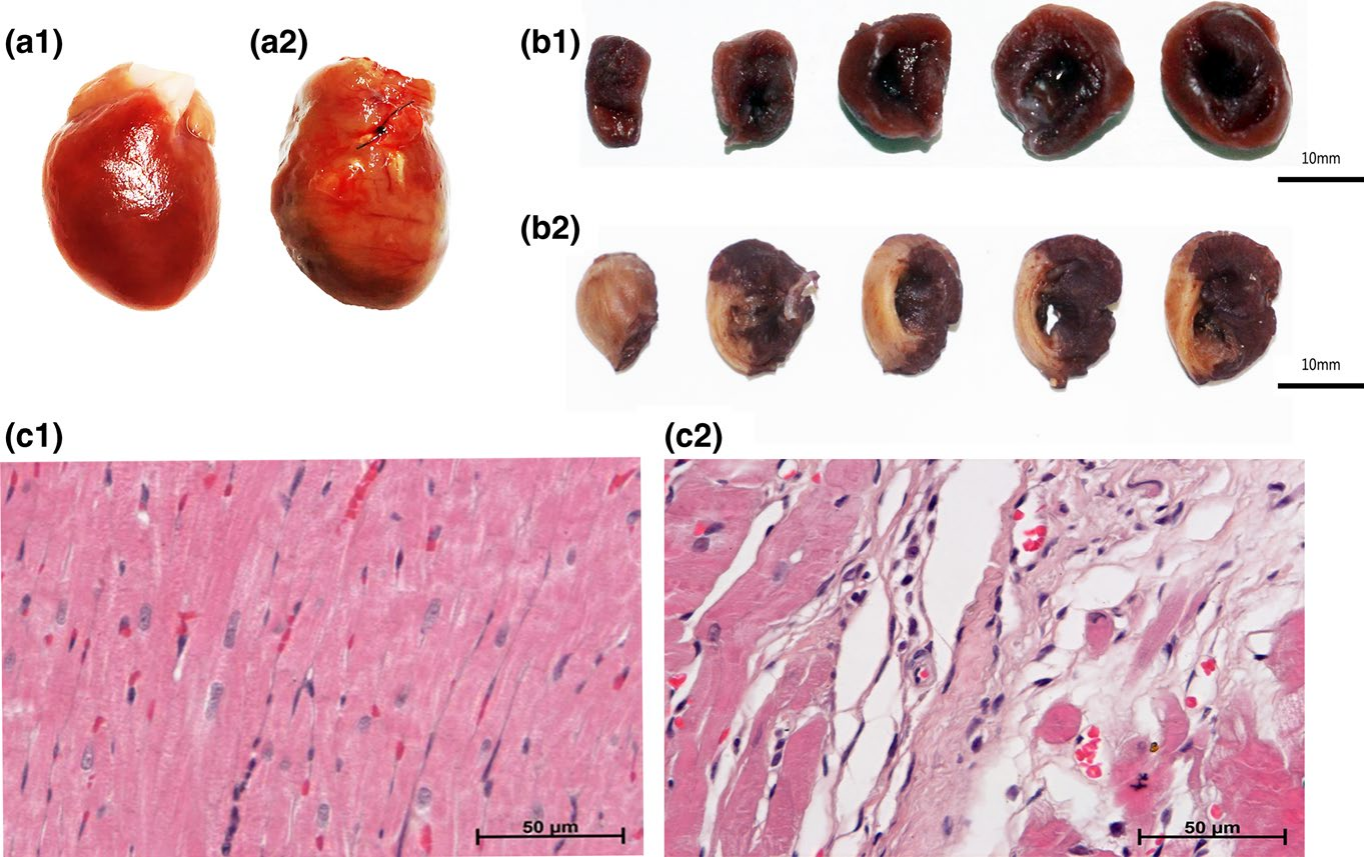
Myocardial histopathological findings. Preparation of the hearts from the sham group (a1) and the model group (a2) after 60 days. The results of NBT staining in the sham group (b1) and the model group (b2). The HE staining results of the sham group (c1) and the model group (c2)

Compared with the sham group, the heart of the model group enlarged and showed significant necrosis of the anterior wall (Figure 2a1,a2).

The results of the NBT staining showed that the myocardia were stained bluish‐purple uniformly in the sham group (Figure 2b1). However, in the model group, lots of gray‐white fibrous tissues without stain were observed in the anterior wall and the anterior septum of the myocardia, indicating the presence of myocardial infarction (Figure 2b2).

#### 3.2.2 Results of HE staining

As shown in Figure 2c1, the myocardial fibers were arranged orderly and tightly with complete structure and clear nucleus boundaries in the sham group. The intercellular substance displayed uniformly, and the cytoplasm was stained evenly. Compared with the sham group, a wide range of necrosis was seen in cardiomyocytes, which were replaced by a lot of fibrous connective tissues. The viable myocardia were disorderly arranged. Nuclear condensation or dissolution and widening of the intercellular substance were also observed in the model group.

The myocardial fibers lost cross striations, and the nuclei were not clearly visible in most of the cells (Figure 2c2).

### 3.3 Results of the Morris water maze and cognitive function of rats with HF

#### 3.3.1 Results of Experiment 1: Acquisition trial, probe trial, and reversal test of Morris water maze

##### Phase 1: Acquisition and Probe trial

**Figure 3:**
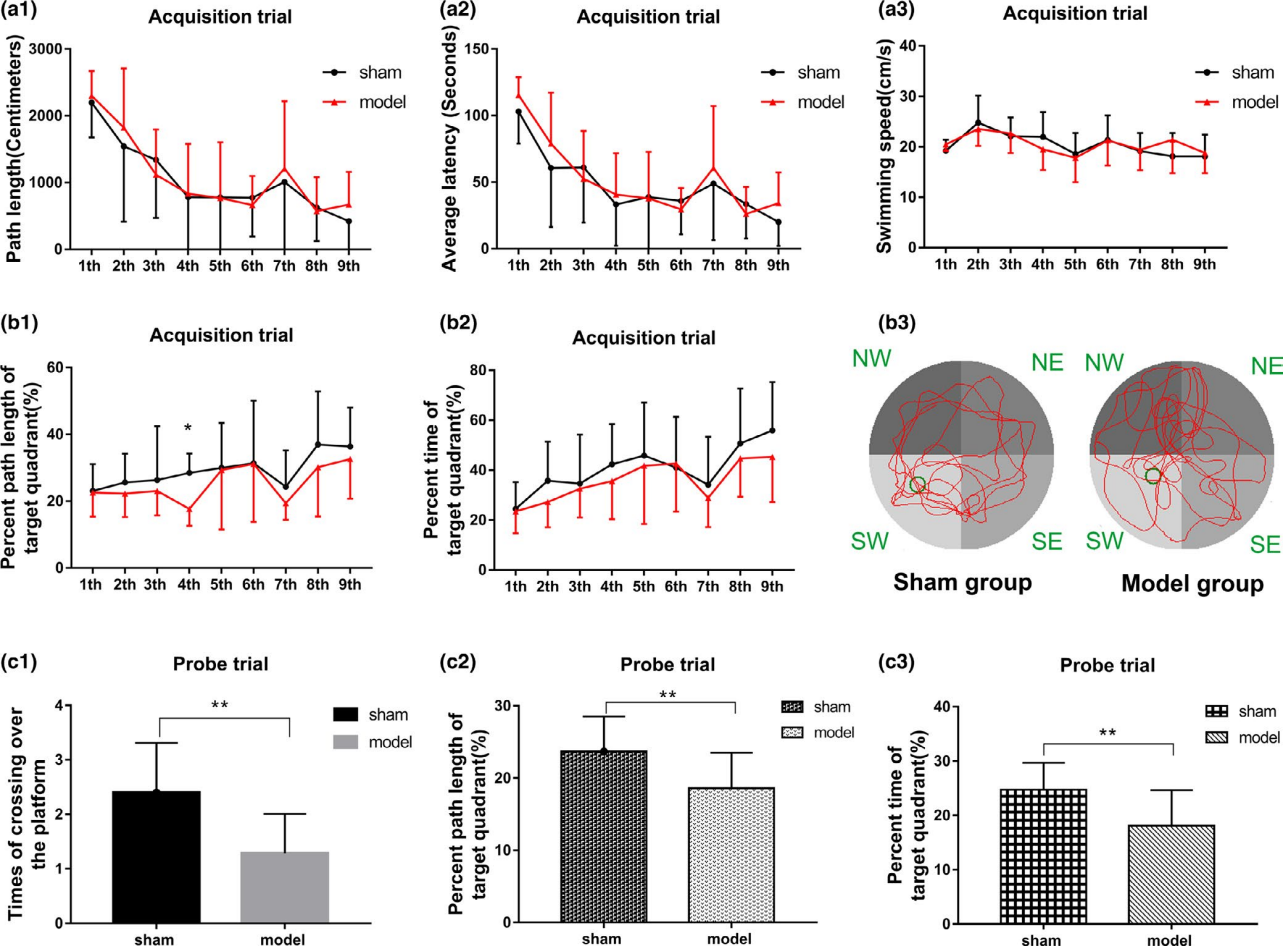
Results of the rats in the acquisition trial and the probe trial of the Morris water maze. The path length of the sham and model groups in the acquisition trial (a1). The average latency of the sham and model groups in the acquisition trial (a2). (a3) Swimming speed of the rats in the sham and model groups in the acquisition trial. The percent path length of the target quadrant between the sham and model groups in the acquisition trial (b1). The percent time of the target quadrant between the sham and model groups in the acquisition trial (b2). (b3) Swimming trajectory images of the sham and model groups. (c1) Number of times the rats in both sham and model groups crossed over the platform in the probe trial. The percent path length of the target quadrant between the sham and model groups in the probe trial (c2). The percent time of the target quadrant between the sham and model groups in the probe trial (C3). *p < .05, **p < .01

In the acquisition trial, no significant difference in path length, average latency, swimming speed, and percent time of the target quadrant (SW) was found between the model and sham groups (Figure 3a1‐a3,b2). However, an obvious decrease in the percentage length of the target quadrant (SW) was noticed in the model group (Figure 3b1, *p* < .05) in the fourth trial.

In the probe trial, when the platform was removed, the number of times the rats in the model group crossed the platform site significantly decreased compared with that in the sham group (Figure 3c1, *p* < .01). Furthermore, the percent path length and time in the target quadrant of model group significantly reduced in probe trial (Figure 3c2,c3, *p* < .01). This evidence indicated that the spatial memory of the model group was impaired.

##### Phase 2: Reversal test

**Figure 4:**
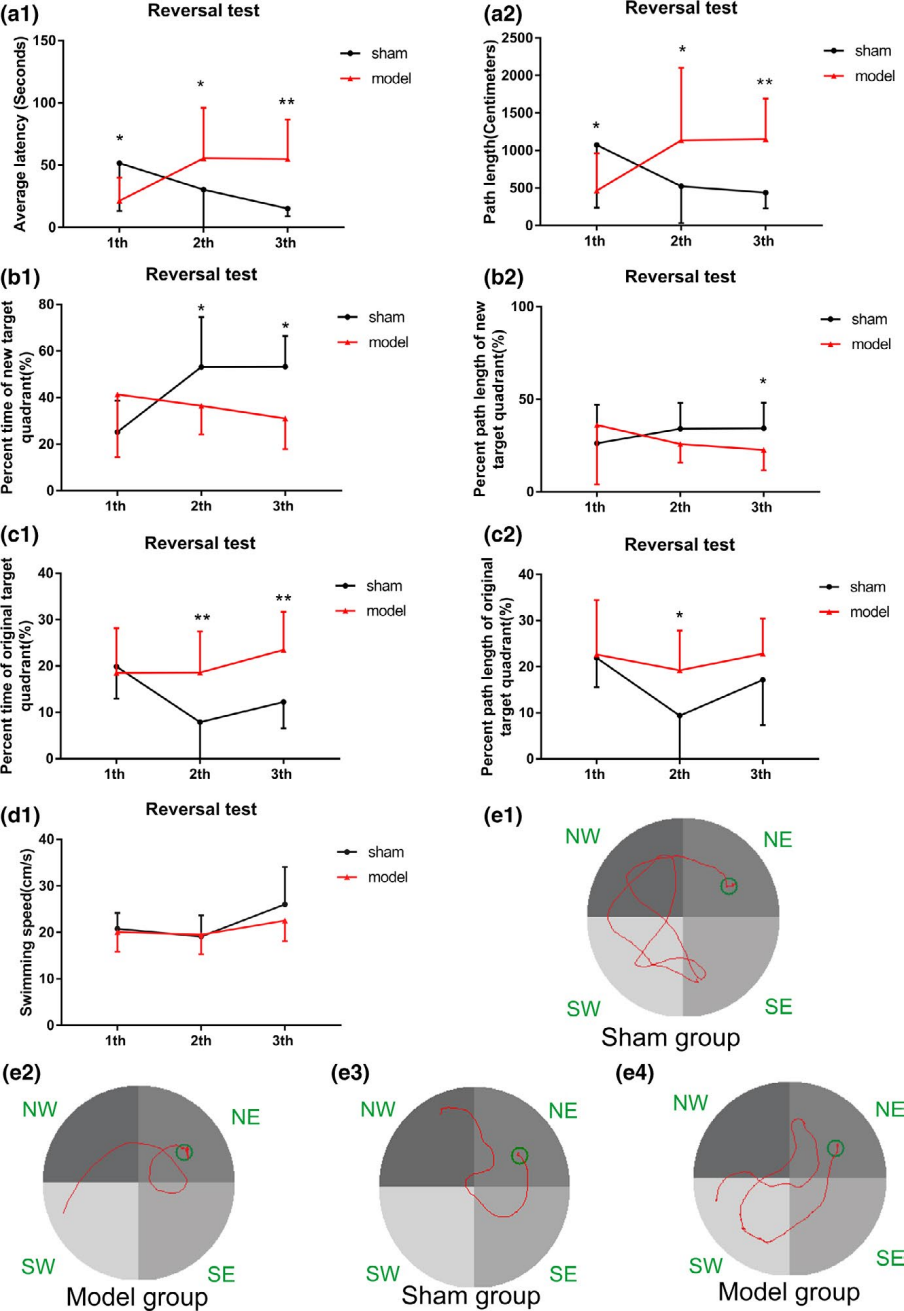
Results of the sham and model groups in the reversal test of the Morris water maze for (a1) average latency, (a2) path length, (b1) percent time of the new target quadrant (NE), (b2) percent length of the new target quadrant (NE), (c1) percent time of the original target quadrant (SW), (c2) percent length of the original target quadrant (SW), and (d1) swimming speed. (e1 and e2) Swimming trajectory images of the sham and model groups in the first, and (e3 and e4) the third trials of the reversal test, respectively. *p < .05, **p < .01

In the reversal test, some changes happened between the model and sham groups. In the first trial, the average latency and swimming path length of the sham group were significantly longer than those of the model group unexpectedly (Figure 4a1,a2; *p* < .05). Further analysis revealed that the percentage time and length in the original quadrant (SW) of the sham group were significantly higher than those in the model group while the results were on the contrary in the new target quadrant (NE; Figure 4b1,b2,c1,c2, *p* < .05), indicating worse memory retrieval of previous information in the model group compared with the sham group. Due to a good memory of the location of the former platform, the rats in the sham group searched for the platform for a significantly longer time in the original target quadrant (SW) and for a shorter time in the target quadrant (NE). However, in the following second and third trials of the reversal test, the average latency and swimming path length of the model group were significantly longer than those in the sham group (Figure 4a1,a2; *p* < .05), and the percent time/length in the new target quadrant (NE) were significantly lower in the model group (Figure 4b1,b2,c1,c2, *p* < .05). These results indicated that the spatial reverse learning and executive function to search for new platforms were impaired in the model group.

#### 3.3.2 Results of experiment 2: Matching‐to‐sample test of the Morris water maze

**Figure 5:**
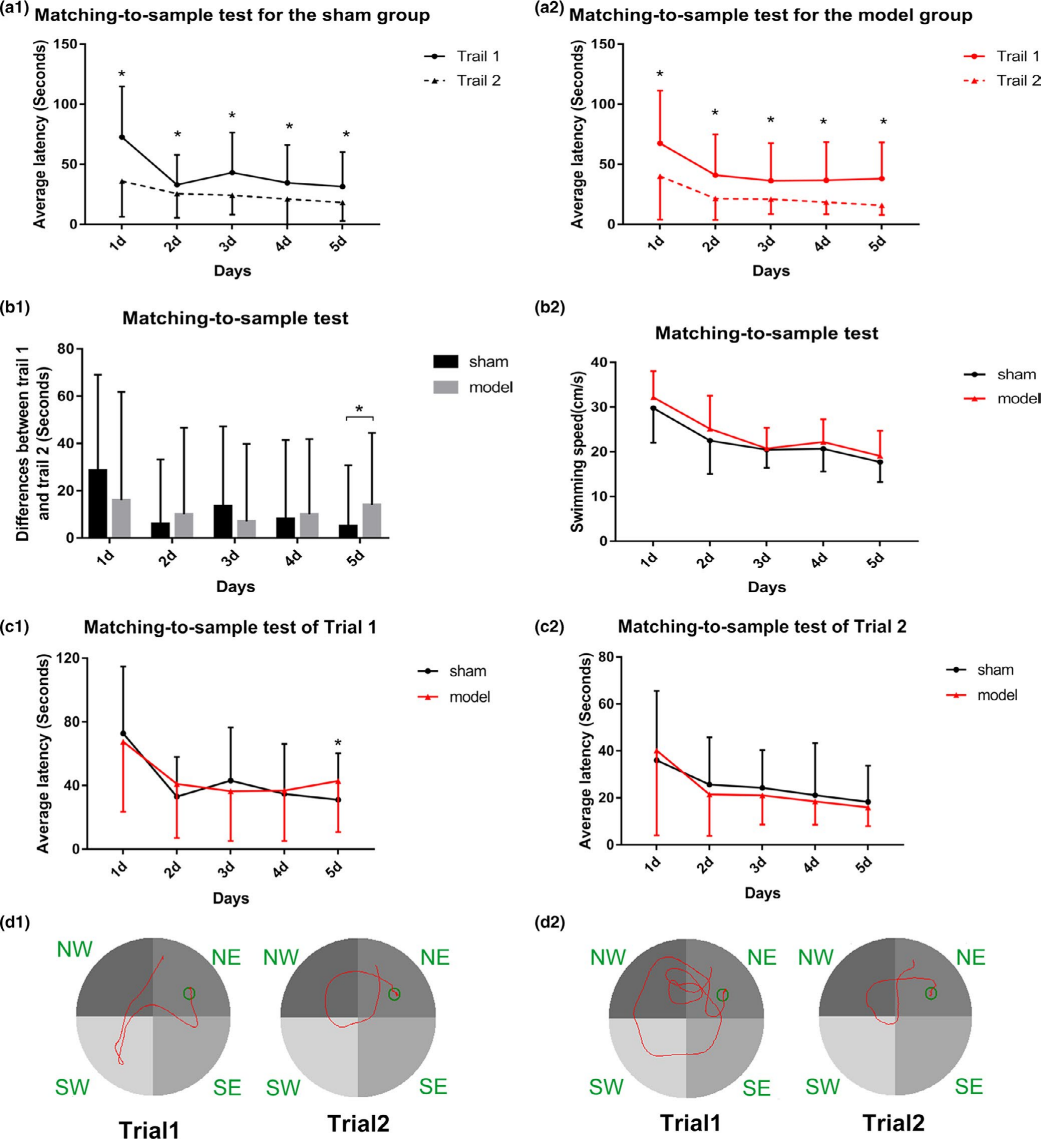
Results of the sham and model groups in the matching‐to‐sample test of the Morris water maze for the average latency of Trial1 and Trial2 (a1) in the sham group and (a2) the model group. (b1) Differences between Trial1 and Trial2 of the sham and model groups for (b2) the swimming speed. (c1 and c2) Average latency of Trial1 and Trial2 between the sham and model groups, respectively. (d1) Swimming trajectory images of the sham group in Trial1 and Trial2. (d2) Swimming trajectory images of the model group in Trial1 and Trial2. *p < .05

To assess the working memory and short‐term memory of the HF model of rats, the matching‐to‐sample test of Morris water maze was used. As shown in Figure 5a1,a2 (*p* < .05), the average latency of Trial2 improved significantly compared with Trial1 in both the model and sham groups, indicating that all the rats were learning the matching‐to‐sample problem and had a memory of the location of the platform in Trial1. No significant differences in the average latency of Trial1 and Trial2 were observed between the model and sham groups from the first day to the fourth day (Figure 5b1). However, unexpectedly, on the fifth day, the difference in the average latency between Trial1 and Trial2 of the model group was significantly larger than that in the sham group (Figure 5b1; *p* < .05). Further analysis found that the average latency of Trial1 increased significantly in the model group compared with the sham group on the fifth day (the average latencies of the sham and model group were 31.02 ± 29.25 s and 42.94 ± 32.19 s, respectively), while no obvious difference in the average latency of Trial2 was found between the model and sham groups, indicating that the rats with HF performed worse in learning the ever‐changing locations of the platform (Figure 5c1,c2; *p* < .05). These pieces of evidence indicated that the short‐term memory of the HF model of rats was not impaired while the executive function to search for a new platform was impaired in the model group.

## 4 DISCUSSION

The early stage of cognitive impairment in HF is characterized by mild cognitive deficits and subtle changes. This study aimed to find a more sensitive behavioral evaluation method to assess cognitive impairment in HF and detect the damage to learning and memory abilities. The spatial acquisition trial, probe trial, reversal task, and matching‐to‐sample test of the Morris water maze were performed to detect the long‐term memory, short‐term memory, and learning of rats with HF in this study. The probe trial and reversal test in the water maze appear to be more sensitive and specific than the matching‐to‐sample test. The cognitive impairments of rats with HF were manifested mainly in spatial memory and learning ability.

In the present study, the HF model of rats was built via ligating left anterior descending coronary artery, which has been widely used in cardiovascular studies (Hou, Huang, Cai, Zhao, & Guo, 2011). The ECG results of the model group showed six to eight obvious pathological Q waves, which were the characteristic diagnostic standard of myocardial infarction (Shettigar, Hultgren, Pfeifer, & Lipton, 1974). Through echocardiography, the EF value of the sham group for 60 days in both experiments 1 and 2 were 81.45% ± 9.79% and 80.12% ± 7.78%, respectively, while in the model group they were 25.68% ± 10.18% and 27.63% ± 5.89%, respectively. This evidence indicated that the HF model of rats was established successfully.

Consistent with the results of ECG and echocardiography, the myocardial histopathological assessment confirmed the myocardial ischemic injury of the model group.

The cognitive impairment in patients with CHF was displayed in different areas of cognitive function, including memory, learning, executive control, attention, processing speed, and fluid intelligence (Hawkins et al., 2012). The characteristics of the cognitive dysfunction in patients with HF are mild and subtle and hence difficult to measure; therefore, different types of cognitive evaluation scales are needed (Pressler, Kim, Riley, Ronis, & Gradus‐Pizlo, 2010; Vogels, Scheltens, Schroeder‐Tanka, & Weinstein, 2014). However, the MMSE and MoCA scales are usually used to evaluate the orientation, memory, visual‐spatial skills, attention, and executive function in patients with HF (Alosco et al., 2013, Dodson, Truong, Towle, Kerins, & Chaudhry, 2013). Furthermore, in clinic, the Wechsler Memory Scale is used as a standardized test for evaluating three different subsystems of memory in patients with HF, including short‐term memory, working memory, and episodic memory (one kind of long‐term memory) (Elwood, 1991; Kindermann et al., 2012; Tulving, 2004). In addition, the assessment of executive function in patients with HF is performed through the Stroop Color Word Interference Task (Dalrymple‐Alford, 1972). Therefore, clinicians may need to use various screening instruments to evaluate the cognitive function of patients with HF more comprehensively. Similarly, in this study, different tasks of the Morris water maze were included to assess the cognition of rats with HF.

The Morris water maze is the most frequently used technique to detect spatial learning, memory, and spatial navigation in laboratory rats with a wide range of application (Brandeis, Brandys & Yehuda, 1989, Hooge & De Deyn, 2001). Moreover, due to the nature of cognitive impairment in HF, the spatial acquisition trial method was not sensitive enough to evaluate cognition successfully. Therefore, different tasks of the Morris water maze were included in this study. In experiment 1, the Morris water maze tests to measure different domains of the cognitive function of rats with HF consisted of three tasks, including the acquisition trial, probe trial, and reversal test. The acquisition and probe trials, typical protocols of the Morris water maze, were used to measure the spatial learning skills and reference memory that was referred to as the long‐term memory in animals, respectively (Mcgarrity, Mason, Fone, Pezze, & Bast, 2016; Sakurai, 1994). The reversal test was used to measure reversal learning, memory, and executive function (Dong et al., 2013). In experiment 2, the matching‐to‐sample test of Morris water maze was used to detect the learning, short‐term memory, or working memory (Frielingsdorf, Thal & Pizzo, 2006) of rats. The reversal test and matching‐to‐sample test were usually used for detecting brain injury in the past. In this study, the matching‐to‐sample test was first used to detect cognitive dysfunction in rats with HF to improve sensitivity and specificity.

In experiment 1, the rats in the model group had significantly impaired memory and learning abilities. No difference in the swimming time and length was observed between the model and sham groups in the spatial acquisition trial. However, in the probe trial, the number of times the rats in the model group crossed the original platform site was significantly less than that in the sham group. This indicated that the long‐term memory of the rats in the model group was seriously damaged. This was consistent with the results that the long‐term memory of patients with HF was worse than that of healthy people in clinical studies (Kindermann et al., 2012). The average latency of the sham group was significantly longer than that of the model group in the first trial of the reverse test, but shorter in the second and third trials. The sham group had a stronger memory of the original target quadrant and thus spent more time searching for the original target quadrant in the first trial (the ratio of the swimming time/path in the original target quadrant to the total swimming time/path increased in the sham group). After learning the new position of the platform, the rats in the sham group spent more time in the new target quadrant and found the platform more quickly in the second and third trials (the ratio of the swimming time/path in the new target quadrant to the total swimming time/path increased in the sham group). This evidence indicated that reverse learning, memory, and executive function of rats in the model group were impaired after 60 days of HF.

In experiment 2, the procedures of the matching‐to‐sample test in the Morris water maze were evaluated in Trial1and Trial2 for each pair of trials (Vorhees & Williams, 2006). The Trial1 was a sample trial designed to let the rats find the platform, while Trial2 represented the matching or memory trial (Serrano Sponton et al., 2018). If the animals recalled the memory of Trial1, they would spend less time to find the platform in Trial2, so that the differences between Trial1 and Trial2 would be larger (Williams et al., 2003). Several studies showed that the average latency of Trial1 had no significant difference between the model and control groups in the rat brain injury model. However, as the model group spent more time to find the goal platform in Trial2, the differences between the trial pairs would be smaller (Frielingsdorf, Thal & Pizzo, 2006, Rodriguez et al., 2018).

However, in this study, the difference in the average latency between Trial1 and Trial2 of the model group was significantly larger than that of the sham group on the fifth day. Further analysis found that the average latency of Trial1 increased significantly in the model group compared with the sham group, while no obvious difference was found in the average latency of Trial2. The explanation of the significantly longer average latency of the model group in Trial1 was that the rats in the model group could not change their search strategy according to the change in the platform location, and its ability to deal with problems decreased. The working memory of the rats with HF may be damaged by affecting the information processing, including encoding, maintenance, and information processing. However, the model group did not spend more time in Trial2 compared with the sham group, indicating that the short‐term memory of the HF model of rats was not impaired seriously. Potential factors contributing to the different results from the previous studies may be mainly the different animal models. In other words, since that the degree and position of brain damage on HF rats were inconsistent with previous studies; therefore, the characteristic of cognitive impairment was also different and so did the results.

However, this study still had some limitations. The language and counting ability of the patients with HF can be detected by screening instrument in clinical studies, which could not be achieved in this study via the animal experiments (Hawkins et al., 2012; Ramos Brieva, Montejo Iglesias, Lafuente, Ponce, & Moreno, 1990).

In summary, the cognitive impairment in rats with HF mainly reflected in the long‐term and working memory, spatial learning, and reversal learning ability. This was consistent with the clinical symptoms of patients with HF. Moreover, we also found that the probe trial and reversal test in the water maze may be more sensitive and preferred although the matching‐to‐sample test can also assess the function of memory. These findings would provide a brief and sensitive behavioral evaluation protocol for further studies on the relationship between cognitive function and HF.

## CONFLICT OF INTEREST

The authors have no competing financial interests to declare.

## AUTHORS' CONTRIBUTIONS

Ziwen Lu and Tao Yang should be considered joint first author. Ziwen Lu and Tao Yang conducted experiments, analyzed data, and wrote the article; Lei Wang, Qi Qiu, Yizhou Zhao,Tong Li, Wenkun Cheng, Baofu Wang, Yang Li, and Jingjing Yang conducted experiments; Aiming Wu contributed reagents/materials/analysis tools; Mingjing Zhao conceived and designed the study, wrote the article, contributed reagents, materials and interpretation of results.

## Data Availability

The data that support the findings of this study are available from the corresponding author upon reasonable request.

## References

[brb31519-bib-0001] Almeida, O. P. , Garrido, G. J. , Beer, C. , Lautenschlager, N. T. , Arnolda, L. , & Flicker, L. (2012). Cognitive and brain changes associated with ischaemic heart disease and heart failure. European Heart Journal, 33(14), 1769–1776. 10.1093/eurheartj/ehr467 22296945

[brb31519-bib-0002] Alosco, M. L. , Brickman, A. M. , Spitznagel, M. B. , Garcia, S. L. , Narkhede, A. , Griffith, E. Y. , … Gunstad, J. (2013). Cerebral perfusion is associated with white matter hyperintensities in older adults with heart failure. Congestive Heart Failure, 19(4), E29–E34. 10.1111/chf.12025 23517434 PMC3692594

[brb31519-bib-0003] Brandeis, R. , Brandys, Y. , & Yehuda, S. (1989). The Use of the Morris Water Maze in the Study of Memory and Learning. International Journal of Neuroscience, 48(1–2), 29–69. 10.3109/00207458909002151 2684886

[brb31519-bib-0004] Cameron, J. , Worrall‐Carter, L. , Page, K. , Stewart, S. , & Ski, C. F. (2013). Screening for mild cognitive impairment in patients with heart failure: Montreal cognitive assessment versus mini mental state exam. European Journal of Cardiovascular Nursing, 12(3), 252–260. 10.1177/1474515111435606 22514141

[brb31519-bib-0005] Dalrymple‐Alford, E. C. (1972). Associative facilitation and interference in the Stroop color‐word task. Perception and Psychophysics, 11(4), 274–276. 10.3758/BF03210377

[brb31519-bib-0006] Davis, K. K. , & Allen, J. K. (2013). Identifying cognitive impairment in heart failure: A review of screening measures. Heart and Lung, 42(2), 92–97. 10.1016/j.hrtlng.2012.11.003 23260324

[brb31519-bib-0007] Dodson, J. A. , Truong, T. N. , Towle, V. R. , Kerins, G. , & Chaudhry, S. I. (2013). Cognitive Impairment in Older Adults with Heart Failure: Prevalence, Documentation, and Impact on Outcomes. The American Journal of Medicine, 126(2), 120–126. 10.1016/j.amjmed.2012.05.029 23331439 PMC3553506

[brb31519-bib-0008] Dong, Z. , Bai, Y. , Wu, X. , Li, H. , Gong, B. , Howland, J. G. , … Wang, Y. T. (2013). Hippocampal long‐term depression mediates spatial reversal learning in the Morris water maze. Neuropharmacology, 64, 65–73. 10.1016/j.neuropharm.2012.06.027 22732443

[brb31519-bib-0009] Elwood, R. W. (1991). The wechsler memory scale—revised: Psychometric characteristics and clinical application. Neuropsychology Review, 2(2), 179–201. 10.1007/BF01109053 1844708

[brb31519-bib-0010] Frielingsdorf, H. , Thal, L. J. , & Pizzo, D. P. (2006). The septohippocampal cholinergic system and spatial working memory in the Morris water maze. Behavioural Brain Research, 168(1), 37–46. 10.1016/j.bbr.2005.10.008 16330106

[brb31519-bib-0011] Hawkins, L. A. , Kilian, S. , Firek, A. , Kashner, T. M. , Firek, C. J. , & Silvet, H. (2012). Cognitive impairment and medication adherence in outpatients with heart failure. Heart and Lung, 41(6), 572–582. 10.1016/j.hrtlng.2012.06.001 22784869

[brb31519-bib-0012] Hong, X. , Bu, L. , Wang, Y. I. , Xu, J. , Wu, J. , Huang, Y. , … Ge, J. (2013). Increases in the risk of cognitive impairment and alterations of cerebral β‐amyloid metabolism in mouse model of heart failure. PLoS ONE, 8(5), e63829 10.1371/journal.pone.0063829 23737953 PMC3667825

[brb31519-bib-0013] Hooge, R. D. , & De Deyn, P. P. (2001). Applications of the Morris water maze in the study of learning and memory. Brain Research Reviews, 36(1), 60–90. 10.1016/S0165-0173(01)00067-4 11516773

[brb31519-bib-0014] Hou, Y. , Huang, C. , Cai, X. , Zhao, J. , & Guo, W. (2011). Improvements in the establishment of a rat myocardial infarction model. Journal of International Medical Research, 39(4), 1284–1292. 10.1177/147323001103900416 21986130

[brb31519-bib-0015] Jennifer, T. (2015). Cognitive impairment predicts worse outcome in heart failure. European Heart Journal, 36(30), 1945. 26478931

[brb31519-bib-0016] Kindermann, I. , Fischer, D. , Karbach, J. , Link, A. , Walenta, K. , Barth, C. , … Böhm, M. (2012). Cognitive function in patients with decompensated heart failure: The cognitive impairment in heart failure (CogImpair‐HF) study. European Journal of Heart Failure, 14(4), 404–413. 10.1093/eurjhf/hfs015 22431406

[brb31519-bib-0017] Langa, K. M. , & Levine, D. A. (2014). The diagnosis and management of mild cognitive impairment: A clinical review. JAMA, 312(23), 2551–2561. 10.1001/jama.2014.13806 25514304 PMC4269302

[brb31519-bib-0018] Mcgarrity, S. , Mason, R. , Fone, K. C. , Pezze, M. , & Bast, T. (2016). Hippocampal neural disinhibition causes attentional and memory deficits. Cerebral Cortex, 27(9), 4447-4462. 10.1001/jama.2014.13806 27550864

[brb31519-bib-0019] O'Donnell, M. , Teo, K. , Gao, P. , Anderson, C. , Sleight, P. , Dans, A. , … Yusuf, S. (2012). Cognitive impairment and risk of cardiovascular events and mortality. European Heart Journal, 33(14), 1777–1786. 10.1093/eurheartj/ehs053 22551598

[brb31519-bib-0020] Pressler, S. J. (2008). Cognitive functioning and chronic heart failure: A review of the literature (2002‐July 2007). Journal of Cardiovascular Nursing, 23(3), 239–249. 10.1097/01.JCN.0000305096.09710.ec 18437066

[brb31519-bib-0021] Pressler, S. J. , Kim, J. , Riley, P. , Ronis, D. L. , & Gradus‐Pizlo, I. (2010). Memory dysfunction, psychomotor slowing, and decreased executive function predict mortality in patients with heart failure and low ejection fraction. Journal of Cardiac Failure, 16(9), 750–760. 10.1016/j.cardfail.2010.04.007 20797599 PMC2929394

[brb31519-bib-0022] Qin, Y. , Wang, S. , Wang, W. , Zhao, M. , LV, X. (2006). Changes of spatial learning and memory ability in rats with heart failure after myocardial infarction. Chinese Journal of Clinical Rehabilitation, 10(2), 172–175.

[brb31519-bib-0023] Ramos Brieva, J. A. , Montejo Iglesias, M. L. , Lafuente, L. R. , Ponce, D. L. H. C. , & Moreno, S. A. (1990). Validation of the Geriatric Depression Screening Scale. Actas luso‐españolas de Neurología, Psiquiatría y Ciencias Afines, 19(3), 174–177. 1950700

[brb31519-bib-0024] Rodriguez, U. A. , Zeng, Y. , Deyo, D. , Parsley, M. A. , Hawkins, B. E. , Prough, D. S. , & DeWitt, D. S. (2018). Effects of Mild Blast Traumatic Brain Injury on Cerebral Vascular, Histopathological, and Behavioral Outcomes in Rats. Journal of Neurotrauma, 35(2), 375–392. 10.1089/neu.2017.5256 29160141 PMC5784797

[brb31519-bib-0025] Sakurai, Y. (1994). Involvement of auditory cortical and hippocampal neurons in auditory working memory and reference memory in the rat. The Journal of Neuroscience, 14(5), 2606–2623. 10.1523/JNEUROSCI.14-05-02606.1994 8182430 PMC6577488

[brb31519-bib-0026] Sauvé, M. J. , Lewis, W. R. , Blankenbiller, M. , Rickabaugh, B. , & Pressler, S. J. (2009). Cognitive impairments in chronic heart failure: A case controlled study. Journal of Cardiac Failure, 15(1), 1–10. 10.1016/j.cardfail.2008.08.007 19181287

[brb31519-bib-0027] Serrano Sponton, L. E. , Soria, G. J. , Dubroqua, S. , Singer, P. , Feldon, J. , Gargiulo, P. A. , & Yee, B. K. (2018). Negative transfer effects between reference memory and working memory training in the water maze in C57BL/6 mice. Behavioural Brain Research, 339, 286–296. 10.1016/j.bbr.2017.10.033 29102592

[brb31519-bib-0028] Shettigar, U. R. , Hultgren, H. N. , Pfeifer, J. F. , & Lipton, M. J. (1974). Diagnostic value of Q‐waves in inferior myocardial infarction. American Heart Journal, 88(2), 170–175. 10.1016/0002-8703(74)90006-4 4841218

[brb31519-bib-0029] Tulving, E. (2004). Episodic memory: From mind to brain. Revue Neurologique, 53(4 Pt 2), S9–S23. 10.1146/annurev.psych.53.100901.135114 15118549

[brb31519-bib-0030] Vogels, R. L. C. , Scheltens, P. , Schroeder‐Tanka, J. M. , & Weinstein, H. C. (2014). Cognitive impairment in heart failure: A systematic review of the literature. European Journal of Heart Failure, 9(5), 440–449. 10.1016/j.ejheart.2006.11.001 17174152

[brb31519-bib-0031] Vorhees, C. V. , & Williams, M. T. (2006). Morris water maze: Procedures for assessing spatial and related forms of learning and memory. Nature Protocols, 1(2), 848–858. 10.1038/nprot.2006.116 17406317 PMC2895266

[brb31519-bib-0032] Williams, M. T. , Morford, L. L. , Wood, S. L. , Wallace, T. L. , Fukumura, M. , Broening, H. W. , & Vorhees, C. V. (2003). DevelopmentalD‐methamphetamine treatment selectively induces spatial navigation impairments in reference memory in the Morris water maze while sparing working memory. Synapse (New York, N. Y.), 48(3), 138–148. 10.1002/syn.10159 12645039

[brb31519-bib-0033] Wu, A. , Zhai, J. , Zhang, D. , Lou, L. , Zhu, H. , Gao, Y. , … Wang, S. (2013). Effect of wenxin granule on ventricular remodeling and myocardial apoptosis in rats with myocardial infarction. Evidence‐Based Complementary and Alternative Medicine, 2013, 1–10. 10.1155/2013/967986 PMC3755410 23997803

[brb31519-bib-0034] Wu, A. , Zhao, M. , Lou, L. , Zhai, J. , Zhang, D. , Zhu, H. , … Chai, L. (2017). Effect of wenxin granules on gap junction and mir‐1 in rats with myocardial infarction. BioMed Research International, 2017, 1–12. 10.1155/2017/3495021 PMC5637836 29094045

